# Alteration of Degree Centrality in Adolescents With Early Blindness

**DOI:** 10.3389/fnhum.2022.935642

**Published:** 2022-06-27

**Authors:** Zhi Wen, Yan Kang, Yu Zhang, Huaguang Yang, Baojun Xie

**Affiliations:** ^1^Department of Radiology, Renmin Hospital of Wuhan University, Wuhan, China; ^2^State Key Laboratory of Magnetic Resonance and Atomic and Molecular Physics, National Center for Magnetic Resonance in Wuhan, Wuhan Institute of Physics and Mathematics, Innovation Academy for Precision Measurement Science and Technology, Chinese Academy of Sciences, Wuhan, China; ^3^University of Chinese Academy of Sciences, Beijing, China

**Keywords:** early blindness, congenital nystagmus (CN), resting-state functional magnetic resonance imaging, degree centrality, neural plasticity

## Abstract

Congenital nystagmus in infants and young children can lead to early blindness (EB). Previous neuroimaging studies have demonstrated that EB is accompanied by alterations in brain structure and function. However, the effects of visual impairment and critical developmental periods on brain functional connectivity at rest have been unclear. Here, we used the voxel-wise degree centrality (DC) method to explore the underlying functional network brain activity in adolescents with EB. Twenty-one patients with EBs and 21 sighted controls (SCs) underwent magnetic resonance imaging. Differences between the two groups were assessed using the DC method. Moreover, the support vector machine (SVM) method was used to differentiate patients with EB patients from the SCs according to DC values. Compared with the SCs, the patients with EB had increased DC values in the bilateral cerebellum_6, cerebellum vermis_4_5, bilateral supplementary motor areas (SMA), and left fusiform gyrus; the patients with EB had decreased DC values in the bilateral rectal gyrus and left medial orbital frontal gyrus. The SVM classification of the DC values achieved an overall accuracy of 70.45% and an area under the curve of 0.86 in distinguishing between the patients with EB and the SCs. Our study may reveal the neuromechanism of neuroplasticity in EB; the findings provide an imaging basis for future development of restorative visual therapies and sensory substitution devices, and future assessments of visual rehabilitation efficacy.

## Introduction

Blindness affects approximately 500 million people worldwide (WHO, 2017). The total number of registered blind people in China is increasing; it has reached 5.5 million. Although 80% of blindness cases involve individuals aged > 50 years, children and young adults who are blind have fewer educational and employment opportunities, lower earning potential, and worse quality of life than individuals who are not blind.

The overall critical period for vision development is from 0 to 3 years of age; the maturation period is from 6 to 8 years of age (Lewis and Maurer, 2009). Specific aspects of vision have different critical periods of development. For example, visual perception of object shape, which is primarily mediated by the ventral visual flow, develops later than visual perception of motion, which is mediated by the dorsal visual flow. Thus, the overall critical period for vision impairment extends until approximately 10 years of age (Bourne and Rosa, 2006).

Early blindness (EB) is defined as visual deprivation before the age of 12, when most children have not completed the critical period for vision development. Visual deprivation can result in reorganization of brain function and structure (Reislev et al., 2016). Compared with the control group, the EB group exhibited significantly less gray matter volume in the visual cortex (Pan et al., 2007), although it exhibited increased cortical thickness in the medial visual cortex (Bridge et al., 2009). Voxel-based morphometry (VBM) and diffusion tensor imaging (DTI) studies revealed that visual tracts and radiations were significantly atrophied in patients with EB (Shimony et al., 2006); longer blindness was associated with more extensive impacts on visual radiations (Ptito et al., 2008). In addition to visual pathway damage, functional changes occur in brain regions secondary to EB. A PET study showed that cerebral blood flow to the cerebellum was significantly increased in patients with EB (Uhl et al., 1993). Functional connectivity among the visual cortex, cerebellum, supplementary motor area (SMA), motor cortex, and temporal cortex was weaker in patients with EB than in controls (Yu et al., 2008; Heine et al., 2015). During auditory processing, the functional connectivity between visual and auditory cortices decreases in patients with EB, while the connectivity the between occipital and temporal cortices increases (Pelland et al., 2017). Overall, vision-related brain regions in patients with EB undergo extensive neuroplasticity to adapt to the disease environment.

Resting-state functional magnetic resonance imaging (MRI) (rs-fMRI) can detect blood oxygen level-dependent changes at rest; the results reflect brain function under physiological and pathological conditions *in vivo*. In the resting state, brain neurons demonstrate a spontaneous activity, which is transmitted to other neurons; the brain thus forms a complex functional network. Degree centrality (DC), which evaluates voxel centrality by assessing the number of connections between that voxel and other voxels at the whole-brain level, can avoid the influence of subjective seed-site selection (Zuo et al., 2012). To some extent, increase or decrease in DC can explain the coordinating and antagonistic effects of brain networks under disease conditions. The DC method has been widely used to analyze ophthalmic diseases such as glaucoma (Cai et al., 2015) and strabismus (Tan et al., 2018). Here, we hypothesized that patients with EB experience visual deprivation-induced reorganization of the brain; the critical developmental period is important for this process. Congenital nystagmus (CN) occurs in infants and young children with no obvious abnormalities in the eyes or the brain. Its main clinical manifestations are involuntary, rhythmic, and reciprocating eye movements and abnormal visual function; it leads to EB. However, the etiology and pathogenesis of CN are unknown. In this study, we performed rs-fMRI to explore the pattern of DC changes in CN. Additionally, we used the support vector machine (SVM) method to investigate the predictive value of DC for clinical diagnosis.

## Materials and Methods

### Participants

This study was approved by the Ethics Committee of Renmin Hospital of Wuhan University. Before undergoing MRI, all the participants or their parents provided written informed consent to participate. Twenty-one patients with EB (10 female patients) and 21 sighted controls (SCs) (11 female patients) aged 15–20 years were enrolled in this study. All the patients with EB were from the School for Blind Children in Wuhan (China); the cause of blindness in all the patients was CN. EB was defined as loss of vision at ≤ 12 years of age. The diagnosis of blindness was made by two experienced ophthalmologists who tested each eye separately and measured visual acuity, visual field, and peripheral vision. Loss of vision was characterized as the inability to see light in either eye.

The inclusion criteria for SCs were as follows: right-handedness, age < 19 years, and binocular vision of ≥ 5. The exclusion criteria for patients with EB and SCs were as follows: lesions visible on MRI (e.g., brain tumors and vascular malformations), history of ocular trauma and/or traumatic brain injury, history of neurological diseases and/or mental illness, and/or contraindications for MRI (e.g., claustrophobia or pacemaker implant).

### Magnetic Resonance Imaging Data Acquisition

MRI scans were performed using a 3.0T MRI scanner (Discovery 750w; GE Healthcare, Milwaukee, WI, United States) with an 8-channel head coil. Foam pads were placed on both sides of the jaw to limit head movements; earplugs were used to reduce exposure to scanning noise. During data acquisition, all the participants were asked to close their eyes, remain awake, and not think about anything specific. rs-fMRI was performed using a gradient-echo planar imaging sequence, and with the following parameters: TR/TE = 2,000/30 ms, flip angle = 90°, FOV = 240 × 240 mm^2^,data matrix size = 64 × 64, slice thickness = 4 mm, interleaved axial slices = 40, and volumes = 210. T1-weighted high-resolution magnetization-prepared rapid gradient-echo structural images were also acquired for alignment and tissue segmentation using the following parameters: TR/TE = 8.5/3.2 ms, flip angle = 9°, matrix size = 256 × 256, voxel size = 1 × 1 × 1 mm^3^, and sagittal slices = 176. T2WI and T2-FLAIR images were collected to exclude participants with brain lesions.

### Magnetic Resonance Imaging Preprocessing

The rs-fMRI data were preprocessed using the Data Processing & Analysis of Brain Imaging (DPABI) toolbox in the MATLAB software (version 2014a). Preprocessing was performed in the following order: (1) data format conversion, (2) removal of the first 10 volumes for each participant, (3) slice timing; (4) head motion correction and acquisition of head motion parameters for each participant, where participants with head movement > 2 mm or rotation > 2° were removed, (5) segmentation, (6) removal of linear drift, (7) filtering, and (8) spatial standardization to Montreal Neurological Institute space with a voxel size of 3 mm × 3 mm × 3 mm.

### Degree Centrality Processing

Using an individual voxel function network, DC was calculated by counting the number of significant suprathreshold correlations (i.e., the degree of binarized adjacency matrix) among the participants. In this study, DC values with a correlation number *r* ≥ 0.25 were calculated using the DPABI software. The following equation was used to convert the voxel-wise DC map of each participant to a z-score map: Zi = DCi meanall/stdall, where Zi is the z-score of a voxel, DCi is the DC value of a voxel, meanall is the average DC value of all voxels in the brain mask, and stdall is the standard deviation of DC values of all voxels in the brain mask.

### Support Vector Machine Analysis

To determine whether changes in DC values could be used as a diagnostic indicator for EB, we performed an ML analysis with the SVM algorithm using the Pattern Recognition for Neuroimaging Toolbox (PRoNTo) software (Schrouff et al., 2013). First, the mean DC values of brain regions that differed between the groups were used as classification features. Then, the leave-one-out cross-validation technique was used to validate the SVM classifier. Accuracy, sensitivity, and specificity were used to quantify the performance of the classification method; receiver operating characteristic curve and corresponding area under the curve values were calculated to assess classification efficacy.

### Statistical Analysis

Two-sample *t*-tests and chi-squared test were performed using the SPSS software (version 22.0) to compare clinical variables between the EB and SC groups. *P*-values < 0.05 were considered statistically significant. Age, sex, and total intracranial volume were used as covariates in a two-sample *t*-test with REST V1.8 (Song et al., 2011) to evaluate differences in voxel-level DC between the groups. The Gaussian random field (GRF) method was used to correct for multiple comparisons (two-tailed, voxel-level *P* < 0.01; GRF correction, cluster-level *P* < 0.05).

## Results

### Demographics and Visual Measurements

There were no significant differences between the EB and SC groups in terms of age (*P* = 0.37), sex (*P* = 0.76), education level (*P* = 0.28), or handedness (*P* = 0.28). The demographic information of the two groups is shown in Table 1.

**TABLE 1:** Demographic information of participants in each group and between-group comparisons.

	EB (*n* = 21)	SC (*n* = 21)	*P*
Age (years)	17.95 ± 1.40	17.76 ± 1.45	0.37
Sex (female/male)	11/10	10/11	0.76
Education level (years)	10.33 ± 1.11	10.76 ± 1.45	0.28
Handedness	21R	21R	1
Duration of blindness (years)	10.14 ± 1.46	−	−
Nystagmus type	18 Vertical/3 horizontal	−	−

*EB, early blindness; SC, sighted controls. Values are expressed as means ± standard deviations or frequencies.*

### Degree Centrality Differences Between Groups

**FIGURE 1:**
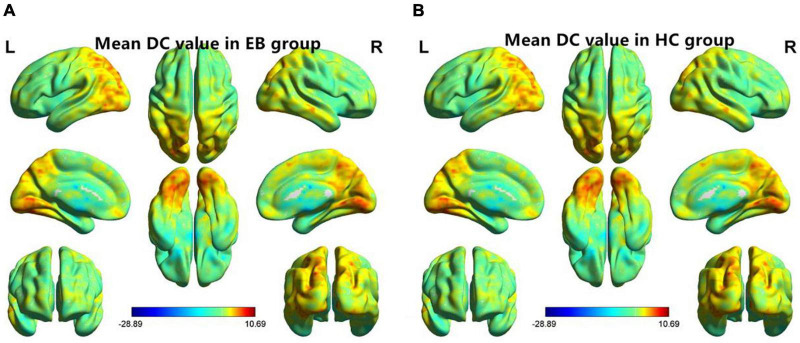
One-sample *t*-test results of maps in the **(A)** EB group and the **(B)** SC group. DC, degree centrality; EB, early blindness; SCs, sighted controls; L, left; and R, right.

**FIGURE 2:**
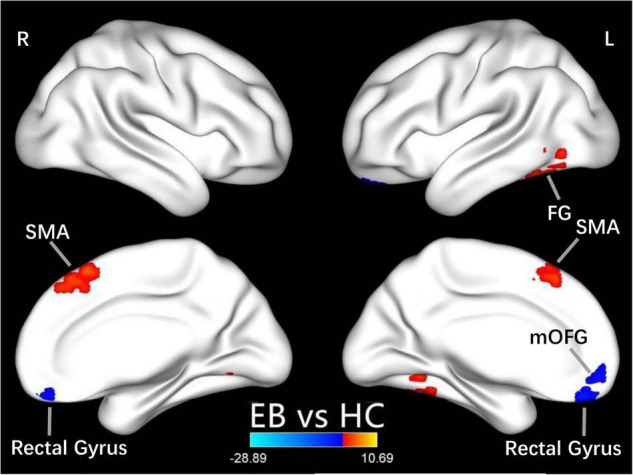
Differences in DC values between the EB and SC groups. Yellow-red denotes higher DC values and green-blue denotes lower DC values in patients with EB compared to SCs. DC, degree centrality; EB, early blindness; SCs, sighted controls; L, left; R, right; FG, fusiform gyrus; SMA, supplementary motor area; and mOFG, medial orbital frontal gyrus.

**FIGURE 3:**
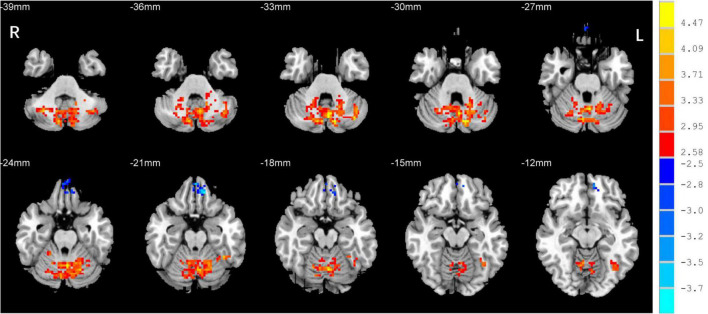
Differences in DC values in the cerebellum between the EB and SC groups. Yellow-red denotes higher DC values and green-blue denotes lower DC values in patients with EB compared to SCs. DC, degree centrality; EB, early blindness; SCs, sighted controls; L, left; and R, right.

One-sample *t*-test was performed to extract the DC values across the subjects within each group (*P* < 0.05; Figure 1). Compared with the SCs, the patients with EB had increased DC values in the bilateral cerebellum_6, cerebellum vermis_4_5, bilateral SMAs, and left fusiform gyrus; they had decreased DC values in the bilateral rectal gyri and left medial orbital frontal gyrus (mOFG) (Table 2 and Figures 2, 3).

**TABLE 2:** Brain regions with significant differences in DC values between the EB and SC groups (Gaussian random field-corrected cluster-level *P* < 0.05).

Specific effects	Identified brain regions	BA	Peak coordinates (MNI)			Side	Peak T	Cluster size (voxels)
			*X*	*Y*	*Z*			
**EB > SC**								
	Supplementary motor area	6	0	24	57	B	4.84	59
	Fusiform gyrus	37	−45	−54	−3	L	3.97	83
	Vermis_4_5	−	0	−63	−6	−	3.56	12
	Cerebellum_6	−	−3	−60	−24	B	4.82	1,099
**EB < SC**								
	Medial orbital frontal gyrus	10	−3	54	−3	L	−3.76	18
	Rectal gyrus	11	−12	42	−21	L	−3.98	20
		11	0	54	−21	B	−3.56	21

*BA, Brodmann area; MNI, Montreal Neurological Institute; EB, early blindness; SCs, sighted controls; L, left; B, bilateral.*

### Support Vector Machine Classification

**FIGURE 4:**
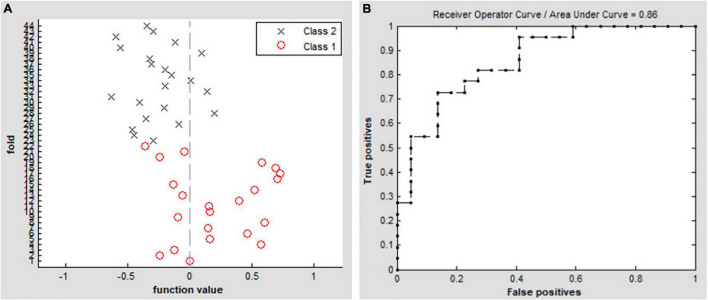
Classification results by SVM analysis based on DC values. **(A)** Functional values in the two groups (class 1: EB group, class 2: SC group). **(B)** ROC curve of the SVM classifier with an AUC value of 0.86. DC, degree centrality; EB, early blindness; SCs, sighted controls; ROC, receiver operating characteristic; SVM, support vector machine; and AUC, area under the curve.

The SVM classification of DC values achieved an overall accuracy of 70.45% and an area under the curve of 0.86 in distinguishing between the patients with EB and the SCs (Figure 4).

## Discussion

In this study, we found that early visual deprivation could cause functional reorganization in several brain regions. Compared with the SC group, the patients with EB had increased DC values in the bilateral cerebellum_6, vermis_4_5, left fusiform gyrus, and bilateral SMAs, while they had decreased DC values in the bilateral rectus gyri and left mOFG. Additionally, using DC as an indicator, the SVM method could distinguish the patients with EB from the SCs with an accuracy rate of 70.45% and an area under the curve of 0.86.

CN first affects patients during childhood. Abnormalities in eye movements and visual perception lead to further compensation by the brain during the development of visual information processing, which leads to increased activation in some brain areas. The cerebellum is responsible for coding and perceiving eye movements (Thier and Markanday, 2019; Lixenberg et al., 2020). Both saccades and smooth tracking are closely associated with the cerebellum (Buttner and Kremmyda, 2007; Quinet et al., 2020). fMRI studies have shown that in patients with CN, activation of the cerebellar vermis increases during horizontal and vertical visual stimulations; thus, the cerebellar vermis inhibits excessive eye movements (Dieterich et al., 2000). A VBM study showed that hypertrophy of the cerebellar vermis occurs in patients with CN because of repeated attempts to inhibit excessive oscillation (Hufner et al., 2011). In contrast, activation of the cerebellar lobe may be primarily associated with changes in attention that maintain eye movement and gaze (Dieterich et al., 2000). In this study, we found increased DC values in the bilateral cerebellum_6 and the cerebellar vermis_4_5 among the *P* patients with EB (Table 2 and Figure 3), which is consistent with the results of previous studies. We presume that the increased DC values in the cerebellum indicate visual and attentional compensations associated with nystagmus.

In our study, compared with the SCs, the patients with EB had increased DC values in bilateral SMAs and decreased DC values in the left mOFG and bilateral rectal gyri (Table 2 and Figure 2). The main cognitive visual pathways include the dorsal and ventral streams (Dutton and Jacobson, 2001). The dorsal stream is responsible for processing the entire input visual scene; this stream includes the occipital, posterior parietal, and frontal cortices. The frontal cortex (including frontal visual areas) produces rapid and accurate eye movements to select targets (Schall, 2004); it is activated at the beginning of eye movements (i.e., voluntary saccades) (Murthy et al., 2007) and pursuit movements (Ono and Mustari, 2009). Motion-related areas receive visual information and generate accurate motion through the visual space. Thus, the frontal eye field, SMA, and medial parietal lobe cooperate during saccade tasks; the frontal eye field, SMA, lateral parietal lobe, and superior/middle temporal gyrus cooperate during pursuit tasks. Our finding that patients with CN had reduced DC values in the left mOFG and bilateral rectal gyri, along with increased DC values in the bilateral SMAs, implies damage to saccadic and pursuit eye movements.

Importantly, we found that DC values in the left fusiform gyrus were higher in patients with EB than in the SCs (Table 2 and Figure 2). The fusiform gyrus is known as the temporo-occipital gyrus; it is involved in object processing in the visual, auditory, tactile modalities, and matching of object-related information across the three sensory modalities (Kassuba et al., 2011). A recent ALE meta-analysis (Zhang et al., 2019) showed that, compared with SCs, persistent activation of the fusiform gyrus in patients with EB was associated with altered object motor function. Therefore, altered DC values in the left fusiform gyrus are associated with object recognition deficits.

There were several limitations in this study. First, the number of patients included was small. However, Friston (2012) suggested that a sample size of > 16 participants per group is acceptable in rs-fMRI studies, consistent with the requirements for a one-sample *t*-test. Our sample sizes were 21 patients with EB and 21 SCs, which exceeded the threshold of > 16 participants per group. Furthermore, we conducted Gaussian random field correction to properly control for false positives; we confirmed the presence of a statistical DC difference between the EB and SC groups. Second, although the ReHo and ALFF methods are reportedly more stable than DC, these methods do not carry any advantage or disadvantage in terms of solving clinical problems. Our study focused on changes in brain network nodes caused by CN; therefore, the DC method was appropriate. Future large-scale studies and research should be conducted from multiple perspectives.

In summary, we found that resting-state DC values were altered among the patients with CN, in association with deficits in saccadic and pursuit eye movements. Our results provide an imaging basis for future development of restorative visual therapies and sensory substitution devices, and future assessments of visual rehabilitation efficacy.

## Data Availability Statement

The original contributions presented in this study are included in the article/supplementary material, further inquiries can be directed to the corresponding author/s.

## Ethics Statement

The studies involving human participants were reviewed and approved by the Ethics Committee of Renmin Hospital of Wuhan University. Written informed consent to participate in this study was provided by the participants’ legal guardian/next of kin.

## Author Contributions

BX designed the study. ZW, YZ, and HY collected the clinical and MRI data. ZW and YK analyzed the MRI data and drafted the manuscript. YK wrote the protocol. HY contributed to the design of the study. All authors contributed to the article and approved the submitted version.

## Conflict of Interest

The authors declare that the research was conducted in the absence of any commercial or financial relationships that could be construed as a potential conflict of interest.

## Publisher’s Note

All claims expressed in this article are solely those of the authors and do not necessarily represent those of their affiliated organizations, or those of the publisher, the editors and the reviewers. Any product that may be evaluated in this article, or claim that may be made by its manufacturer, is not guaranteed or endorsed by the publisher.
